# Put MY mask on first: Mothers’ reactions to prioritizing health behaviours as a function of self-compassion and fear of self-compassion

**DOI:** 10.1177/1359105321995979

**Published:** 2021-02-18

**Authors:** Kaeley M Simpson, Brittany N Semenchuk, Shaelyn M Strachan

**Affiliations:** University of Manitoba, Canada

**Keywords:** beliefs, health promotion, norms, postpartum, women’s health

## Abstract

Self-compassion predicts mothers’ engagement in health behaviours yet some mothers fear self-compassion. We examined relationships between both self-compassion and fear of self-compassion with mothers’ reactions to prioritizing health behaviours. Mothers rated their self-compassion, fear of self-compassion, read a scenario about prioritizing health behaviours and rated adjectives describing themselves if they behaved as described in the scenario. Self-compassion was positively related to positive reactions to engaging in health behaviours in the midst of motherhood. Fear of self-compassion led to negative reactions. This research provides insight into why some mothers feel better about incorporating health behaviours into their lives than others.

The motherhood transition presents demands (Sevón, 2012) and stress (Baxter et al., 2008) that can challenge mothers to balance motherhood with other roles (e.g., work, relationship with partner) (Delicate et al., 2018; Sevón, 2012). Mothers often set aside their needs in favour of their children’s (Martinez et al., 2011; Miller and Brown, 2005) and report having to make many personal sacrifices due to motherhood role demands (Moore and Abetz, 2019). This tendency may be reflected in mothers’ diminished engagement in health behaviours (HBs) upon having children (McGannon and Schinke, 2013). Physical activity, healthy eating and sleep are key to health (Bauman, 2004; Pepin et al., 2014) yet mothers engage in HBs less frequently than women without children (Bellows-Riecken and Rhodes, 2008).

Barriers to HBs among mothers include lack of time (Berge et al., 2011), work (Fahrenwald and Shangreaux, 2006) and family demands (Mailey et al., 2014). The expectation that motherhood should be prioritized can also be a barrier to mothers’ engagement in HBs (McGannon and Schinke, 2013). Through socialization, women learn that ‘good mothers’ prioritize the needs of others’ (Gilligan, 1982). Additionally, studies show that individuals are likely to act according to behaviours they deem to be morally relevant (Christner et al., 2020). That is, if women believe prioritizing the needs of their children is what is morally correct, they are more likely to act accordingly. Engaging in HBs requires mothers take time away from being ‘mom’, which can lead to guilt (Martinez et al., 2011) and feelings of being a ‘bad mother’ (McGannon and Schinke, 2013).

Although many mothers experience guilt when taking time away from their children (Martinez et al., 2011), some believe engaging in HBs helps them be a good parent (Mailey et al., 2014; Palmer and Leberman, 2009). Some mothers navigate the guilt for taking time to engage in HBs through integrating HBs into their lives (Mailey et al., 2014; Palmer and Leberman, 2009). In light of the benefits of HBs (Chaput et al., 2014), and the drop in participation among mothers with young children (Bellows-Riecken and Rhodes, 2008), it is important to understand factors that influence mothers to favourably view engaging in HBs in the midst of motherhood. Self-compassion (SC) may be one such variable.

Self-compassion consists of self-kindness, mindfulness and common humanity (Neff, 2003a). Self-kindness involves extending warmth to oneself during difficult times rather than self-criticism (Neff et al., 2020). Mindfulness is holding one’s suffering in balanced awareness, with neither rumination or avoidance (Neff et al., 2020). Common humanity involves the recognition that one is not isolated in their suffering (Neff et al., 2020). Self-compassion is related to indicators of health and well-being including positive affect, happiness and optimism (Neff and Vonk, 2009) and helps individuals adaptively cope with negative emotions and challenges (Neff and Germer, 2017).

Through mitigating negative and promoting positive feelings about focusing on one’s own health and well-being (Neff, 2003a), SC should help mothers engage in HBs. Self-compassion is associated with lower feelings of shame and guilt during parental challenges (Sirois et al., 2018). A meta-analysis by Sirois et al. (2015) found an association between SC and low negative affect, and the relationship between SC and high positive affect, predicted engagement in HBs. Among new mothers who had become less active, SC was associated with positive reactions including less shame about their reduced physical activity and mothers reported that their physical activity created less conflict with their motherhood role (Kullman et al., 2019). Miller and Strachan (2020) found that mothers’ SC was associated with lower guilt about taking time away from their family to engage in HBs, which was in turn associated with HBs.

Despite this promising research, some people are hesitant to extend themselves compassion, because they fear it will make them self-indulgent (Neff et al., 2007). This fear of SC is associated with self-criticism, depression and anxiety (Gilbert et al., 2011). The ethics of dominant societal expectations of mothers (Gilligan, 1982; McGannon and Schinke, 2013) may make fear of SC prevalent among mothers who feel it is wrong to take time away from their motherhood role (Miller and Brown, 2005).

Through engaging in HBs and positively impacting their health, mothers engage in a self-compassionate act (Miller and Strachan, 2020). However, little is known about how mothers feel about prioritizing their health in the midst of motherhood. The purpose of this study is to determine how SC and fear of SC relate to mothers’ feelings about prioritizing their needs to engage in HBs. Based on previous research, we hypothesize that SC will be associated with positive assessments, and fear of SC will be associated with negative assessments of taking time to engage in HBs. Through examining these relationships, we will better understand how SC and fear of SC relate to mothers’ feelings about prioritizing their health. This improved understanding will inform SC intervention efforts with mothers of young children who engage in significantly less HBs once they become mothers (Bellows-Riecken and Rhodes, 2008; Berge et al., 2011).

## Method

### Design and participants

According to G* Power, a sample of 85 was required to achieve 80% power (α = 0.00278) with an expected effect size of *r* = −0.40 between SC and guilt about engaging in HBs (Miller and Strachan, 2020). A Bonferroni correction set our alpha at 0.00278. Upon ethics approval, 111 mothers completed our cross-sectional study online through Prolific Academic. Eligibility required being over the age of 18, ability to read English and having at least one child under the age of 5.

### Procedures

Participants consented and completed demographic information, study scales and a parenting scenario task and then were debriefed and compensated with $3.88 CAD.

### Measures

#### Demographics

Participants reported their age, cultural background, number of children, childrens’ ages, marital and employment status, education background and country of residence.

#### Self-compassion scale

Self-compassion was assessed using the 26-item SC Scale (Neff, 2003b). This scale assesses the three components of SC and their opposing constructs: self-kindness (self-criticism), mindfulness (over-identification) and common humanity (isolation) using a 5-point Likert scale ranging from 1 (*almost never*) to 5 (*almost always*). Negatively worded items were reversed-scored and a grand mean was calculated.

#### Fear of self-compassion

Fear of SC was measured using the 15-item Fear of Compassion for Self Scale (Gilbert et al., 2011), which employs a 5-point Likert scale ranging from 0 (*don’t agree at all*) to 4 (*completely agree*). All items were summed and then a mean was calculated.

#### Parenting scenario and bipolar adjective scale

This scenario assessed participants’ feelings about being self-compassionate through prioritizing their engagement in HBs. The scenario read as followed:

*As a mom you have many responsibilities. You take on a large caregiving role for your child(ren) and find value in caring and looking after them. As a mother you recognize that in order to meet the needs of your children you must also take care of your own needs. Although it may be difficult to find time for yourself, you work hard to make time to engage in health-promoting behaviours such as exercise, good diet, and sleep. You know it is best to prioritize your own personal needs in order to be able to best support and care for your children.*

After imagining the scenario participants rated self-perceptions if they thought, felt and behaved as described in the scenario using 17 bipolar adjectives (e.g., confident-insecure, unhappy-happy, admirable-shameful). Participants responded using a 7-point Likert scale ranging from −3 to +3, with endpoints labelled ‘very’. For example, participants could rate themselves as ‘very confident’ (−3) or ‘very insecure’ (+3). The midpoint of zero is labelled as ‘neither’. This methodology was used previously by Robinson et al. (2016).

### Data sharing statement

The current article includes the complete raw data-set collected in the study including the participants’ data set, syntax file and log files for analysis. Pending acceptance for publication, all of the data files will be automatically uploaded to the Figshare repository. The data is stored on a spreadsheet and contains participant demographics, SC (highlighted in yellow), fear of SC (green) and bipolar adjective ratings (orange). The attached SPSS result outputs contain the results from the Bivariate Pearson correlations that were conducted in the present study. The supplemental tables include participant demographics and descriptives for each scale used.

### Analysis

Using IBM SPSS Statistics 26.0 the relationship between SC and fear of SC with each of the bipolar adjectives were assessed using a series of Pearson bivariate correlations. Prior to our analysis, the data was cleaned and examined according to recommendations made by Field (2017). There were no violations of normality or univariate outliers within the data.

### Participant description

Participant’s (*n* = 111) age ranged from 19 to 59 years with a mean age of 32.52 (SD = 6.57). Most women had two children (51.3%), lived in Europe (79.1%), were Caucasian (73.9%), completed some university education (46.8%), worked full time (58.5%) and were married (70.2%). Further demographics can be found in Supplemental Table 1.

### Results

Descriptives for variables along with scale reliabilities can be found in Supplemental Table 2. Pearson correlations revealed SC was significantly related to 10 of 17 bipolar adjectives. Higher trait SC scores were associated with higher scores on feeling: like a success, confident, industrious, relaxed, happy, modest, responsible, strong, admirable and ambitious when imagining behaving in the way the scenario described. The relationships between SC and the remaining seven bipolar adjectives were not significant (see Table 1).

**Table 1.** Pearson correlation analyses: bipolar adjectives with self-compassion and fear of self-compassion.

Variable	Self-compassion	Fear of self-compassion
Bipolar adjectives		
Confident versus insecure	−0.25**	0.30**
Lazy versus industrious	0.20*	−0.17
A success versus a failure	−0.28**	0.30**
Anxious versus relaxed	0.31**	−0.27**
Unhappy versus happy	0.34**	−0.35**
Responsible versus irresponsible	−0.19*	0.29**
Self-centred versus other-oriented	0.04	−0.04
Pleasant versus unpleasant	−0.13	0.28**
Arrogant versus modest	0.25**	−0.28**
Careful versus careless	−0.05	0.19*
Judgemental versus non-judgmental	0.08	−0.23*
Competent versus incompetent	−0.17	0.29**
Weak versus strong	0.32**	−0.22*
Admirable versus shameful	−0.31**	0.30**
Ambitious versus non-ambitious	−0.22*	0.18
Competitive versus cooperative	0.10	−0.14
Self-indulgent versus self-denying	−0.11	0.30**

*Note.* *p < 0.05. ** *p* < 0.01.

Fear of SC was positively related to 13 of 17 bipolar adjectives. Participants who scored high on fear of SC reported higher scores on feeling: like a failure, insecure, anxious, unhappy, irresponsible, unpleasant, arrogant, careless, judgmental, incompetent, weak, shameful and self-denying when imagining behaving in the way the scenario described. The relationships between fear of SC and the remaining four bipolar adjectives were not significant (see Table 1).

## Discussion

One-way mothers of young children can be self-compassionate, despite the demands of motherhood, is through prioritizing time for HBs. Self-compassion relates to mothers’ engagement in HBs (Miller and Strachan, 2020), yet little is known about how mothers feel about offering themselves compassion; the present study filled this gap. Self-compassion was generally associated with positive feelings about prioritizing HBs and fear of SC was associated with participants feeling more negative.

### Self-compassion and bipolar adjectives

A well-documented challenge mothers experience is balancing the often-conflicting roles in motherhood (Choi et al., 2005). Despite mothers having personal needs, women are socialized to learn that the needs of others should be prioritized above their own (Govrin, 2014), which can lead to the experience of challenging emotions when taking time away from their children (Borelli et al., 2016; Milke et al., 2015). In the present study, SC was associated with mothers feeling more positively when imagining prioritizing HBs. This finding suggests the extent to which mothers are self-compassionate can influence how they feel about making time to engage in HBs. Robinson et al. (2016) found similar results among participants recruited through a psychology subject pool; self-compassionate individuals reported feeling more relaxed and happier when imaging behaving in self-compassionate ways. Self-compassion is linked to people pursuing what is best for their health and well-being (Terry and Leary, 2011; Zessin et al., 2015). According to self-determination theorists, engaging in a behaviour for these reasons should lead to positive outcomes such as behavioural adherence (Teixeira et al., 2012). Our findings underscore this idea among mothers of young children; mothers who are self-compassionate rate that they would feel, for example, ‘relaxed and strong’ if they prioritized HBs. Self-compassion may work to buffer messages around prioritization of others’ needs that mothers often learn through socialization, that may serve as a barrier to HBs. Our results suggest that SC may be an effective way to increase positive emotions mothers experience when prioritizing HBs.

### Fear of self-compassion and bipolar adjectives

Fear of SC was associated with negative feelings about taking time to engage in HBs. Previous research shows that some people fear SC, believe they do not deserve to be self-compassionate and prefer self-criticism (Joeng et al., 2015). Individuals that fear SC often see being self-critical (as opposed to self-compassionate) as a way to be accountable and take responsibility (Robinson et al., 2016). Our results align with these ideas as mothers high in fear of SC rated feeling, for example ‘irresponsible and incompetent’ when imagining engaging in HBs. The negative feelings mothers fearful of SC experience when imagining taking time away from their children may make them less likely to engage in HBs. Therefore, although SC may help mothers of young children feel more positively about prioritizing HBs, guarding against fear of SC is also important.

### Strengths and implications

A strength of our study is that our sample included mothers from a variety of countries with various employment statuses, educational backgrounds and a large age range. However, the majority of mothers in our sample lived in Europe and were Caucasian. Therefore, our sample is generalizable on some aspects but lacks diversity and has limited generalizability to non-Caucasian populations. Through this study we expand our understanding of how mothers with young children feel about being self-compassionate through engaging in HBs. Research has established a link between SC and mothers’ self-reported engagement in HBs (Dunne et al., 2016), but to our knowledge, no study has specifically examined how mothers feel about prioritizing their own needs to engage in these HBs. These findings identify variables through which SC may lead to HBs. For example, the finding that SC is associated with mothers feeling ‘confident and ambitious’ suggests that variables such as self-efficacy may link SC to HBs among mothers. Such a possibility cannot be confirmed in this study as we did not measure HB engagement. This proposed sequence should be explored in future prospective or experimental research. The findings are also insightful for the development of interventions within this population.

An implication of this research is that SC training should be incorporated into pre and postnatal care. Training depressed pregnant women in SC led to better well-being post-partem (Guo et al., 2020); doing so may also make mothers more apt to view engagement in HBs favourably. Given fear of SC is associated with feelings that may make engagement in HBs psychologically uncomfortable for women (e.g., irresponsible), it would also be important to address these fears in interventions with mothers. Interventions could include various forms of compassion focused therapy incorporating psychoeducation on SC, understanding barriers and fears to the development of SC, emotional regulation and strategies for reducing self-criticism. Such SC interventions are shown to be effective in improving the self-regulation of health behaviours (Biber and Ellis, 2019). Some mothers view engaging in HBs as key to being a good mother (Mailey et al., 2014; Palmer and Leberman, 2009), therefore, educating mothers about how extending themselves SC may position them to better care for others (Neff and Beretvas, 2013; Neff and Pommier, 2013) may assuage fear of SC.

### Limitations and future directions

This study is limited by its correlational design; causal inferences cannot be made. Future researchers should examine the relationship between SC and dependent variables within a prospective or an experimental design. In addition, for some participants the relationship between SC and reactions using the bipolar adjective scale may have been influenced by how they felt about engaging in HBs. Future researchers should consider participants’ desires surrounding engagement in HBs.

## Conclusion

Our results suggest that SC may help mothers of young children positively view the prioritization of HBs during the busy and stressful time of motherhood, while mothers who fear self-compassion may feel negatively about this idea. These findings add to previous knowledge about factors that may promote (and discourage) engagement in HBs among mothers with young children and suggest that promotion of SC pre- and post-natally should be examined for its potential benefit.

## Supplemental Material

sj-pdf-1-hpq-10.1177_1359105321995979 – Supplemental material for Put MY mask on first: Mothers’ reactions to prioritizing health behaviours as a function of self-compassion and fear of self-compassion Click here for additional data file. Supplemental material, sj-pdf-1-hpq-10.1177_1359105321995979 for Put MY mask on first: Mothers’ reactions to prioritizing health behaviours as a function of self-compassion and fear of self-compassion by Kaeley M Simpson, Brittany N Semenchuk and Shaelyn M Strachan in Journal of Health Psychology
