# Supplementary material for: Put MY mask on first: Mothers’ reactions to prioritizing health behaviours as a function of self-compassion and fear of self-compassion
Source: J Health Psychol. 2021 Feb 18;27(5):1259–66. doi: 10.1177/1359105321995979 (PMC8978476; doi:10.1177/1359105321995979)
Supplement: sj-pdf-1-hpq-10.1177_1359105321995979 – Supplemental material for Put MY mask on first: Mothers’ reactions to prioritizing health behaviours as a function of self-compassion and fear of self-compassion [file sj-pdf-1-hpq-10.1177_1359105321995979.pdf]

Supplemental Table 1

Participant Demographics (n = 111)

Variable		*N*	%
Age			
	19 – 30	47	42.3
	31 – 40	51	45.9
	41 – 50	12	10.8
	51 – 60	1	0.9
Cultural Background			
	Caucasian	82	73.9
	Indigenous	0	0
	Hispanic	5	4.5
	African American	2	1.8
	Asian	7	6.3
	Other	15	13.5
Education Completed			
	High School	16	14.4
	College/Vocational School	26	23.4
	University	52	46.8
	Postgraduate	17	15.3
Employment Status	Stay at home mother	23	20.7
	Student	3	2.7
	Part-time	20	18.0
	Full-time	65	58.5
Marital Status			
	Single	14	12.6
	Common-law	16	14.4
	Married	78	70.2
	Separated/Divorced	3	2.7
Number of Children	1	41	36.9
	2	57	51.3
	3	7	6.3
	4	3	2.7
	5+	3	2.7

Supplemental Table 2

Descriptive Statistics

Variable	Range	M	SD	Alpha
Self-Compassion	1.35 – 4.50	2.84	0.65	0.92
Fear of Self-Compassion	0 – 3.40	1.44	0.82	0.92
Confident vs Insecure	-3 – 3	-0.71	1.65	N/A
Lazy vs Industrious	-2 – 3	1.01	1.41	N/A
A Success vs A Failure	-3 – 3	-1.02	1.56	N/A
Anxious vs Relaxed	-3 – 3	-0.04	1.76	N/A
Unhappy vs Happy	-3 – 3	1.17	1.41	N/A
Responsible vs Irresponsible	-3 – 3	1.45	1.62	N/A
Self-centered vs Other-oriented	-3 – 3	0.16	1.39	N/A
Pleasant vs Unpleasant	-3 – 2	-1.46	1.23	N/A
Arrogant vs Modest	-2 – 3	0.96	1.29	N/A
Careful vs Careless	-3 – 2	-1.58	1.26	N/A

Judgmental vs Non-judgmental	-3 – 3	-0.03	1.34	N/A
Competent vs Incompetent	-3 – 2	-1.47	1.16	N/A
Weak vs Strong	-2 – 3	1.04	1.38	N/A
Admirable vs Shameful	-3 – 3	-0.98	1.24	N/A
Ambitious vs Non ambitious	-3 – 2	-1.14	1.28	N/A
Competitive vs Cooperative	-3 – 3	0.19	1.55	N/A
Self-indulgent vs Self-denying	-3 – 2	-0.65	1.17	N/A
